# Proteome microarray-guided identification of mycobacterial antigens and ELISA-based peptide mapping for improved serological detection of *Mycobacterium bovis* infection in European badgers

**DOI:** 10.1128/jcm.01260-25

**Published:** 2026-01-27

**Authors:** Gareth A. Williams, Sabah Rahou, Ollie Bateman, Andy A. Teng, Angela Yee, Joseph J. Campo, Laura Arnold, Richard J. Delahay, Thomas Holder, Dipesh Davé, Mark A. Chambers, H. Martin Vordermeier

**Affiliations:** 1 Department of Bacteriology, Animal and Plant Health Agency (APHA) 16232, Addlestone, United Kingdom; 2 Antigen Discovery Inc. (ADI), Irvine, California, USA; 3 National Wildlife Management Centre, Animal and Plant Health Agency (APHA) 16232, York, United Kingdom; University of Western Australia, Perth, Western Australia

**Keywords:** *Mycobacterium bovis*, bovine tuberculosis, badger, tuberculosis, serodiagnosis, Rv3616c, proteome microarray, epitope mapping

## Abstract

Bovine tuberculosis, a zoonotic disease caused primarily by *Mycobacterium bovis*, poses a significant threat to cattle health and farming livelihoods within the United Kingdom (UK). Disease control in cattle is complicated by the persistence of *M. bovis* in European badgers, the UK’s principal wildlife reservoir. Accurate diagnostic tools for both species are essential for effective surveillance and disease control. Many existing badger serodiagnostic tests, which include MPB70, MPB83, and ESAT6-CFP10 antigens, have relatively modest sensitivities (~50%–60%), limiting their utility in surveillance. To address this issue, we used an unbiased and comprehensive antigen discovery approach to identify new diagnostic targets. This strategy identified Rv3616c as a novel antigen with promising diagnostic test potential for *M. bovis* infection in badgers. Overlapping peptides spanning the full Rv3616c amino acid sequence were screened to identify the most diagnostically informative epitopes. A pool of four Rv3616c peptides, used in an indirect enzyme-linked immunosorbent assay (ELISA), had a sensitivity of 85.71% (95% CI: 77.19–91.96), a specificity of 94.80% (95% CI: 90.35–97.59), and a diagnostic accuracy of 91.51% (95% CI: 87.54–94.54). The existing validated Badger *M. bovis* Ab Test, when used alone, had a sensitivity of 73.47% (95% CI: 63.59–81.88); however, parallel interpretation with the Rv3616c ELISA could increase overall sensitivity to 91.84% (95% CI: 84.55–96.41), with minimal loss of specificity. These findings support the use of Rv3616c-derived peptides in serodiagnostic tests to improve the detection of *M. bovis* infection in badgers and enhance tuberculosis surveillance in this wildlife reservoir.

**IMPORTANCE:**

Accurate diagnosis of *Mycobacterium bovis* infection in wildlife reservoirs is essential for controlling bovine tuberculosis (bTB), a zoonotic disease that threatens human health, animal welfare, and farming livelihoods. In the United Kingdom, European badgers are the principal wildlife reservoir, complicating efforts to eradicate bTB in cattle. Existing serodiagnostic tests for badgers have moderate sensitivity, limiting effectiveness in surveillance. To address this, this study used an unbiased, comprehensive antigen discovery approach and identified several new diagnostic targets, including the Rv3616c protein. A test based on specific Rv3616c-derived peptides had a high diagnostic accuracy (91.51%) and, when used in parallel with a validated test, improved test sensitivity while maintaining specificity. These synthetic peptides are scalable, cost-effective, and adaptable to different diagnostic platforms. The findings reveal an antigen with diagnostic potential that could inform the development of new tests for bTB surveillance in wildlife, supporting One Health principles and global tuberculosis elimination strategies.

## INTRODUCTION

Bovine tuberculosis (bTB) is a zoonotic disease that has a significant negative impact on global human and animal health, and is endemic in many low- and middle-income countries (1–3). *Mycobacterium bovis* (*M. bovis*) is the primary etiological agent of bTB in cattle and represents an ongoing threat to the livelihoods of cattle farmers and farming communities (4–6). Within the United Kingdom (UK), eradication of bTB is hampered by *M. bovis* infection in European badgers (*Meles meles*), creating a badger-cattle disease episystem with complex transmission dynamics (7–10). Despite this complexity, and as part of the UK Government’s long-term bTB eradication strategy, the use of enhanced disease control measures in cattle and targeted reductions in badger density (badger culling) have, in recent years, been associated with a decrease in bTB in geographical areas of England with a relatively high disease incidence (11, 12). *M. bovis* Bacillus Calmette-Guérin, Danish strain 1331 (BCG) vaccination of badgers has been shown to be effective at reducing the severity and progression of disease (13) and the spread of infection (14), with wider deployment in the field expected in the coming years (15). In the future, BCG vaccination may also provide a safe additional bTB control measure for cattle (16–19) but would not necessarily prevent infection or onward transmission in all vaccinated animals (20). Consequently, the ongoing development and refinement of disease control measures and surveillance strategies for both cattle and badgers will need to be maintained if the goal of bTB eradication is to be achieved (21). Selecting the most appropriate measures for controlling *M. bovis* infection in cattle and badgers for specific epidemiological situations and assessing the impact of these on disease incidence and prevalence are critically dependent on the accurate diagnosis of infection in both hosts (22, 23).

Diagnostic tests for tuberculosis (TB) can be broadly categorized into those that detect the presence of *M. bovis* bacilli or its constituent parts (such as microscopy, microbiological culture, and PCR) in tissues or clinical samples collected from animals, and those that detect an infected animal’s immune response (such as tuberculin skin testing and blood-based immunodiagnostic assays) elicited following exposure to *M. bovis* (24). The former test category is most typically used for postmortem TB infection status confirmation, genetic typing, and infection tracing, and the latter for primary TB diagnosis and disease surveillance. Cost-effective and technically undemanding immunological tests, such as individual lateral-flow cassette-format serological tests that can be used to diagnose TB infection in live animals (point-of-capture tests [POCT]), often provide the greatest utility for disease surveillance in wildlife species (25). Laboratory-based diagnostic tests, such as enzyme-linked immunosorbent assays (ELISAs), are, however, more suited to the larger sample sizes required for seroprevalence investigations in wildlife. The range and performance of immunology-based TB diagnostic tests for badgers and other wildlife species have been the subject of recent comprehensive reviews (22, 26).

Commercial serological tests that detect specific antibody responses to *M. bovis* infection, such as the BrockTB STAT-PAK (now discontinued), DPP VetTB Assay (Chembio Diagnostic Systems, Inc.), and the IDEXX *M. bovis* Ab Test (IDEXX Laboratories Ltd.) have been used in operational TB surveillance and in captive and wild badger research studies (27–31). These tests utilize existing and extensively characterized mycobacterial antigens such as MPB70, MPB83, ESAT-6, and CFP-10 (22). Apart from the Badger *M. bovis* Ab Test (a modified version of the IDEXX test; sensitivity [Se] 72%) and an encouraging report of an ELISA based on a P22 target antigen (Se 74%–82%) (32), many serological diagnostic tests developed for badger TB typically have relatively modest sensitivities (~50%–60%) (22, 33), possibly due to the limited number of antigens used (34). The P22 complex is a mixture of at least 10 proteins immunopurified from bovine tuberculin with a relatively high Se for a badger TB diagnostic test (35); however, the number of proteins involved may make it more challenging to manufacture a P22-based diagnostic test to an acceptable standard at commercial scale. To date, no comprehensive assessment has been carried out to identify other badger antigens that might enable either more sensitive serological tests to be developed or tests suitable for use in a POCT format.

Technology developed by Antigen Discovery Inc. (ADI, Irvine, CA) enables high-throughput expression of genomic open reading frame (ORF) libraries and printing of their protein products as individual spots onto nitrocellulose microarrays. Such microarrays can be probed with antibody-rich biological samples (e.g., sera) to identify antigens with diagnostic potential. ADI’s infectious microorganism library collection includes the genome of *M. tuberculosis* H37Rv (MTb H37Rv), which is >99.95% identical to the genome of *M. bovis* (36) and includes the RD1, RD2, and RD3 genes present in *M. bovis* but absent in the BCG strain used for badger vaccination (37–39). As a proxy for the *M. bovis* proteome, the MTb H37Rv proteome microarray library was therefore mined in this study for potential novel diagnostic antigenic targets recognized by badger antibodies using a panel of serum samples collected from badgers of known TB infection status.

One of the key antigens identified using ADI’s proteome microarray technology was the protein product of the *Rv3616c* gene (ESX-1 secretion-associated protein A or EspA), which was available to us as a pool of overlapping synthetic peptides spanning the full amino acid sequence encoded by *Rv3616c*. The serodiagnostic potential of this Rv3616c overlapping synthetic peptide pool was assessed using an in-house badger ELISA and serum samples from TB-free and TB-infected badgers. The performance of a badger ELISA containing selected peptides from the Rv3616c peptide pool was compared to that of the Badger *M. bovis* Ab Test, which has been developed and validated at the Animal and Plant Health Agency (APHA) as a badger TB diagnostic test. This comparison identified Rv3616c-derived peptides as novel diagnostic antigens with the potential to improve TB diagnostic testing performance in badgers.

## MATERIALS AND METHODS

### Study objectives

The overarching objective of this study was to identify novel serodiagnostic antigens for *M. bovis* infection in badgers using a proteome microarray discovery approach. The serum panel used for this contained samples from badgers with defined TB infection and BCG vaccination statuses, enabling a broad preliminary screen of serum antibody reactivity. Among the antigens identified, Rv3616c was selected for further investigation based on the results of this initial screen and the availability of overlapping synthetic peptides. In summary, the study progressed through a series of connected exploratory phases, each informed by the results of the preceding phase. These phase-specific objectives are summarized below and are reflected in the organization of the Results section:

- Antigen discovery: detection of antigens with serodiagnostic potential using a proteome microarray screening approach.
- Epitope mapping and peptide-based ELISA development: identification of immunodominant epitopes within the Rv3616c antigen for inclusion in a peptide-based ELISA (Rv3616c-4P Badger ELISA).
- Diagnostic evaluation: determination of potential test cut-off values and an assessment of the diagnostic performance of the Rv3616c-4P Badger ELISA.
- Comparative testing: evaluation of the diagnostic performance of the Rv3616c-4P Badger ELISA in comparison with the validated Badger *M. bovis* Ab Test.
- Parallel testing: assessment of the impact of parallel testing using both the Rv3616c-4P Badger ELISA and the Badger *M. bovis* Ab Test on overall Se and specificity (Sp).

### Provenance of badger serum samples

No animal procedures were performed during this study. All serum samples were obtained from archived collections originating from unrelated scientific studies carried out by the APHA. These samples were promptly frozen at –80°C and stored in small-volume aliquots to minimize potential antibody degradation from freeze–thaw cycles. Although no new animal procedures were carried out in this study, detailed provenance information is provided for all archived serum samples used, in line with best practices for transparent reporting of research involving animal-derived materials.

#### TB-free samples

Archived serum samples had been collected from badgers sourced from geographic areas of the UK with no reported cases of TB in either cattle or badgers. During these historical studies, the animals were housed in APHA facilities, and appropriate biosecurity measures were taken to prevent the introduction of any potential sources of TB infection. A TB-free status had been assigned to the animals following three consecutive (carried out at approximately 1-month intervals) negative badger interferon-gamma release assay test results (40). Additionally, *M. bovis* had not been cultured from any tracheal or rectal swab samples collected from these animals up to and including the time of blood sample collection (41).

#### TB-infected samples—experimental infection

Archived serum samples had been collected from captive TB-free badgers following their experimental infection with 10^3^ colony-forming units (CFU) of *M. bovis* via the endobronchial route (42). TB infection in these animals had been confirmed by the presence of postmortem macroscopic pathology and histopathological examination results characteristic of TB infection, and by isolation of *M. bovis* from postmortem tissue samples (43).

#### TB-infected samples—natural infection

Archived serum samples from badgers naturally infected with TB had been collected from an intensively studied wild population located in Woodchester Park, Gloucestershire, UK. This study area of approximately 7 km^2^ contains about 20 badger social groups, which have been part of a long-term capture-mark-recapture study (44). For this study, TB-infected animals were defined as those that had a positive *M. bovis* microbiological culture result from any clinical sample (such as urine, feces, tracheal/esophageal aspirates, and bite wound swabs). Such a result indicated the presence of an active infection and bacterial shedding, thus identifying animals of epidemiological importance (45).

#### BCG-vaccinated samples

Archived serum samples had been collected from captive TB-free badgers that had received two separate doses of 2–8 × 10^6^ CFU BadgerBCG vaccine (AJ Vaccines, Copenhagen, Denmark) by intramuscular injection. Samples had been collected 12 months after the primary vaccination with BCG (1 × BCG) and then again 4 weeks after the repeat/booster BCG vaccination (2 × BCG), administered 12 months after the primary vaccination.

### Badger serum panel used to mine the MTb H37Rv proteome microarray

The badger serum panel used to mine the MTb H37Rv proteome microarray was assembled from sera collected from 35 different badgers and comprised 99 individual samples from animals of differing TB infection and BCG vaccination status. It included 87 individual serum samples from 29 different badgers, collected at three discrete time points: before experimental infection with *M. bovis*, and at 8 and 12–13 weeks after infection (immediately before euthanasia and postmortem examination). The serum panel also included an additional 12 samples collected from six different TB-free badgers after they had received a primary BCG vaccination and again after a booster BCG vaccination administered 12 months later (these animals were not experimentally infected with *M. bovis*).

### Antigen mining of the MTb H37Rv proteome microarray

ADI constructed an MTb H37Rv proteome microarray containing approximately 4,100 full-length or fragmented proteins, collectively representing approximately 99% of the MTb H37Rv protein-coding genome. Proteome microarrays were fabricated using partial or complete ORFs cloned into the T7 expression vector pXI (ADI, Irvine, CA, USA) via a high-throughput PCR recombination cloning method. All MTb H37Rv ORFs were cloned and sequenced, and sequencing results confirmed the correct target identity for all clones.

Proteins were expressed using an *Escherichia coli*-based cell-free *in vitro* transcription/translation (IVTT) system (biotechrabbit, Berlin, Germany) with 5′ polyhistidine and 3′ hemagglutinin tags, according to the manufacturer’s instructions. Translated proteins and IVTT controls were printed onto custom nitrocellulose-coated microarray slides (Grace Bio-Labs, Bend, OR, USA) using a GeneMachines OmniGrid Accent Microarray Printer. Badger sera were diluted 1:100 and incubated overnight with the MTb H37Rv proteome microarray slides. Serum antibody binding was detected using a fluorescently labeled secondary antibody. Microarray slides were then scanned using a high-resolution GenePix 4300A Microarray Scanner (Molecular Devices, Sunnyvale, CA, USA), and signals were quantified using Mapix software (Innopsys, Carbonne, France).

### Rv3616c synthetic peptides

**Fig 1:**
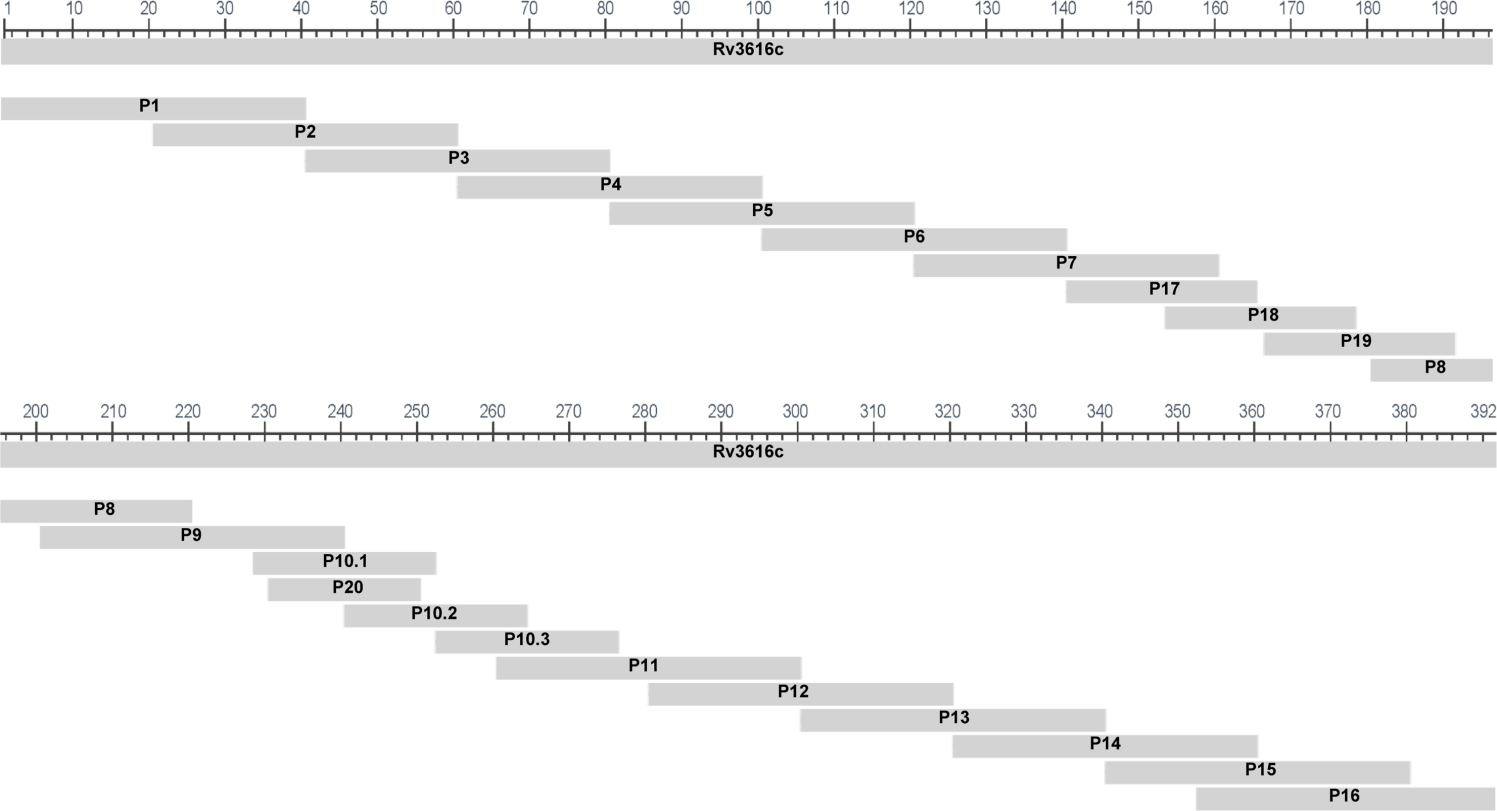
Mapping of overlapping synthetic peptides with the *M. tuberculosis* H37Rv Rv3616c amino acid sequence. The horizontal scale indicates amino acid positions in the Rv3616c protein (ESX-1 secretion-associated protein A, the *M. tuberculosis* H37Rv *espA* gene product). Twenty-two overlapping peptides (20–40 amino acids in length), labeled “P” followed by their unique identification numbers, are aligned with the Rv3616c sequence. P8 spans both the upper and lower areas of the figure layout but represents a single peptide.

The Rv3616c protein was produced by GenScript Biotech (Piscataway, NJ, USA) in the form of 22 overlapping purified synthetic peptides, each 20–40 amino acids in length, that collectively spanned the entire 392-amino acid sequence of Rv3616c. A schematic alignment of the 22 overlapping peptides with the Rv3616c protein sequence is shown in Fig. 1, and peptide amino acid sequences are provided in Table S1.

### Badger *M. bovis* Ab Test

The Badger *M. bovis* Ab Test was carried out as described in the manufacturer’s instructions for the IDEXX *M. bovis* Ab Test Kit (IDEXX Laboratories Ltd., Wetherby, UK) used to detect antibodies (to *M. bovis)* present in cattle serum or plasma samples, but with two minor modifications: (i) badger TB-positive and TB-negative sera internal quality controls were used in duplicate, in addition to the commercial kit’s bovine positive and negative controls; and (ii) the commercial kit’s anti-bovine IgG-horseradish peroxidase conjugate antibody detection solution was replaced with an anti-badger IgG-horseradish peroxidase conjugate solution (CF2-HRP, a custom APHA reagent) at a concentration of 1 µg/mL in 1% (wt/vol) BSA-PBS for any wells that contained badger samples (i.e., badger test sera and badger internal quality controls). On completion of the testing protocol, the final absorbance of each well at 450 nm was measured using a Multiskan FC Microplate Photometer (Thermo Fisher Scientific, Loughborough, UK), and the resulting data were collected by SkanIt Software for Microplate Readers (Thermo Fisher Scientific). The test cut-off value for this APHA-validated diagnostic test for TB in badgers is >1.674.

### Rv3616c Badger ELISA

F96 PolySorp Nunc-Immuno plates (Thermo Fisher Scientific) were coated with Rv3616c synthetic peptides, 50 µL/well at a concentration of 5 µg/mL in 0.1 M carbonate coating buffer (pH 9.6) for 16 hours at 2°C–8°C. The carbonate coating solution was then discarded by decanting and repeatedly tapping inverted plates onto an absorbent paper towel. Each well then received 200 µL of blocking solution (4% [wt/vol] BSA in PBS), and the plates were incubated for 1 hour at 20°C–25°C. Excess blocking solution was discarded, and the plates were then washed six times with PBS containing 0.5% (vol/vol) Tween-20. Serum samples to be tested and APHA badger TB positive and TB negative internal quality controls were diluted 1:50 in the same blocking solution, 50 µL of each was then added to the plate wells, and the plates were incubated at 20°C–25°C for 1 hour. The plates were then washed as previously described. Each well then received 50 µL of CF2-HRP in PBS containing 1% (wt/vol) BSA, and the plates were incubated for a further hour at 20°C–25°C. Excess CF2-HRP solution was discarded, the plates were washed as previously described, and then 50 µL of the substrate 3,3′,5,5′-tetramethylbenzidine (TMB; Sigma-Aldrich, Gillingham, UK) was added to each well. Plates were incubated for 10 minutes at 20°C–25°C after which any ongoing oxidation of TMB was stopped by the addition of 50 µL of 0.5 M sulfuric acid to each plate well. The absorbance of each well at 450 nm was then measured using a Multiskan FC Microplate Photometer (Thermo Fisher Scientific), and the resulting data were collected by SkanIt Software for Microplate Readers (Thermo Fisher Scientific).

### Rv3616c synthetic peptide ELISA screening

A primary screen of the 22 individual synthetic peptides, collectively spanning the Rv3616c amino acid sequence, was carried out by coating separate ELISA plates with each individual peptide and carrying out the Rv3616c Badger ELISA using the 29 TB-free and 29 TB-infected (12–13 weeks post-infection) badger serum samples that were submitted as part of the proteome microarray antigen mining serum panel. ELISA-based screening of Rv3616c peptides for immunodominant diagnostic epitopes ultimately identified four peptides (Peptides 10.2, 12, 13, and 15) that had the greatest potential ability to differentiate TB-free and TB-infected badger serum samples. These four peptides were subsequently combined and used as a pooled coating antigen (5 µg/mL per peptide) in a single ELISA (referred to as the Rv3616c-4P Badger ELISA) to test serum samples originally collected from 173 TB-free and 98 TB-infected badgers (26 experimentally infected and 72 naturally infected). Serum sample test results from experimentally and naturally infected animals were combined into a single TB-infected group for analysis purposes, unless otherwise stated.

### Statistical methods

Statistical analyses for the proteome microarray antigen mining phase were carried out using R (http://www.R-project.org). All other statistical analyses were performed using GraphPad Prism 8 (version 8.4.2; GraphPad Software, Bishop’s Stortford, UK) and the MedCalc Diagnostic Test Evaluation Calculator (version 23.3.5; https://www.medcalc.org/calc/diagnostic_test.php).

#### Identification of antigens with serodiagnostic potential

Raw proteome microarray signal intensities were log₂-transformed and background-corrected by subtracting the median signal intensity of the IVTT control spots for each sample, providing a relative measure of specific versus nonspecific antibody binding to the IVTT expression system (signal-to-noise ratio). Paired *t*-tests were carried out on log₂-transformed and background-corrected fluorescence intensity data for comparisons between TB-free (*n* = 29) and TB-infected badgers at 8 weeks (*n* = 29) and 12–13 weeks post-infection (*n* = 29). Independent *t*-tests were used to compare results for TB-infected badgers at 12–13 weeks post-infection (*n* = 29) with BCG-vaccinated animals that received one dose (1 × BCG, *n* = 6) or two doses (2 × BCG, *n* = 6). *P*-values were adjusted using the Benjamini-Hochberg (BH) procedure to control the false discovery rate resulting from multiple comparisons. Differences were considered statistically significant when BH-adjusted *P* < 0.05. Antigens were subsequently ranked by *t*-test BH-adjusted *P*-values (smallest to largest). Fold-change values in mean spot intensities were derived from exponentiated log₂-transformed values.

#### ELISA screening of Rv3616c peptides to identify immunodominant diagnostic epitopes

ELISA absorbance fold-change values were calculated by dividing the mean absorbance obtained for TB-infected samples (12–13 weeks post-infection; *n* = 29) by the mean absorbance of TB-free samples (*n* = 29) for each of the 22 Rv3616c synthetic peptides. Receiver operating characteristic (ROC) analyses were used to derive Se, Sp, and the area under the curve (AUC) was calculated using the Hanley-McNeil method (46).

#### ROC analysis and cut-off value determination for the Rv3616c-4P Badger ELISA

Rv3616c-4P Badger ELISA absorbance values obtained for TB-free (*n* = 173), experimentally TB-infected (12–13 weeks post-infection; *n* = 26), and naturally TB-infected (*n* = 72) badger serum samples were compared using a nonparametric ANOVA (Kruskal-Wallis test), followed by Dunn’s multiple comparison post hoc analysis. Se, Sp, and AUC were determined as previously described, and test accuracy was calculated as (Se × Pr) + (Sp × (1 − Pr)), where Pr was the infection prevalence represented by the test sample set. Test cut-off optimization methods for diagnostic performance metrics comparisons included: Euclidean Distance (cut-off at minimum √[(1 − Se)² + (1 − Sp)²]); Youden’s J Statistic (cut-off at maximum Se + Sp − 1); Maximum Product (cut-off at maximum Se × Sp); Index of Union (cut-off at minimum (Se − AUC) + (Sp − AUC)); and Diagnostic Odds Ratio (DOR; cut-off at maximum (Se × Sp)/[(1 − Se) × (1 − Sp)]).

#### Comparative performance of the Rv3616c-4P Badger ELISA and the Badger *M. bovis* Ab Test

Diagnostic performance of the different tests was assessed using serum samples collected from TB-free badgers (*n* = 173) and TB-infected badgers (*n* = 98). Se, Sp, AUC, and accuracy were determined as previously described. The DOR was calculated as the ratio of the positive likelihood ratio to the negative likelihood ratio. Test performance metrics were compared using McNemar’s test for paired proportions and a *z*-test applied to the natural log-transformed DOR values.

#### Diagnostic performance of parallel testing with the Badger *M. bovis* Ab Test and Rv3616c-4P Badger ELISA

Parallel testing performance was evaluated by classifying samples (TB-free, *n* = 173; TB-infected, *n* = 98) as true positives if either the Badger *M. bovis* Ab Test or the Rv3616c-4P Badger ELISA returned a positive result and as true negatives only if both tests classified samples as negative. A fixed cut-off of >1.674 was used for the Badger *M. bovis* Ab Test as this is an APHA-validated diagnostic test for TB in badgers. For the Rv3616c-4P Badger ELISA, the potential cut-off values derived from the test cut-off optimization methods for stand-alone testing (i.e., >0.1072, >0.1279, >0.2328, >0.2689, and >0.2737) were re-evaluated to assess the effect of each threshold on overall test performance in a parallel testing context.

## RESULTS

### Identification of antigens with serodiagnostic potential

**Fig 2:**
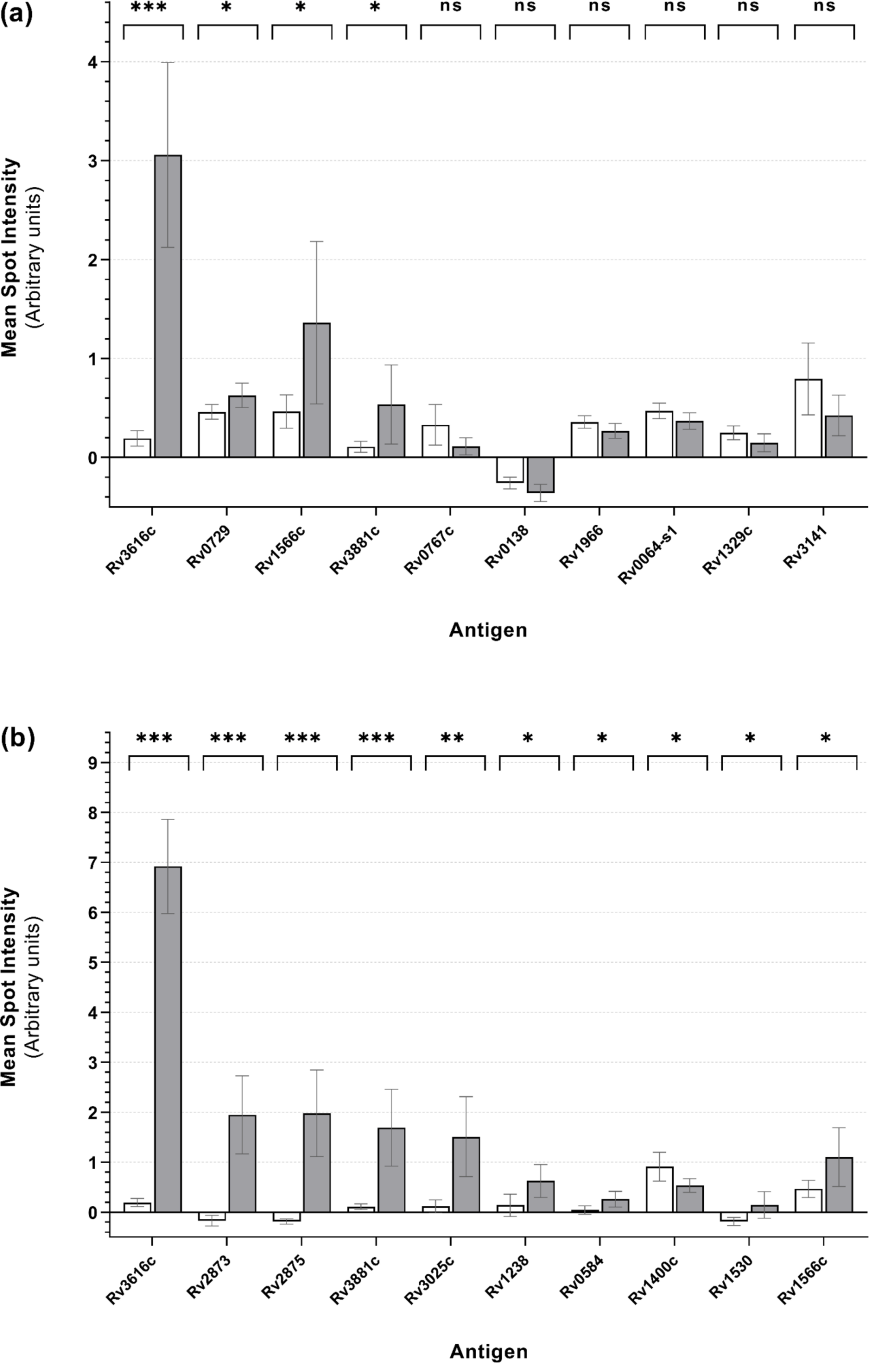
Mean microarray antigen spot intensities from TB-free badger serum samples at two time points following experimental infection. White bars represent TB-free sera; gray bars represent TB-infected sera collected at (**a**) 8 weeks and (**b**) 12–13 weeks post-infection. Mean microarray spot intensities (log_2_-normalized) are shown with 95% confidence interval error bars. Paired-sample *t*-tests were carried out comparing pre- and post-infection mean spot intensities. Antigens were ranked by BH-adjusted *P*-values, and the 10 most differential antigens (defined as those with the lowest BH-adjusted *P*-values) for each comparison group are shown. Significance levels: ns: *P* ≥ 0.05; *: *P* < 0.05; **: *P* < 0.01; and ***: *P* < 0.001.

**Fig 3:**
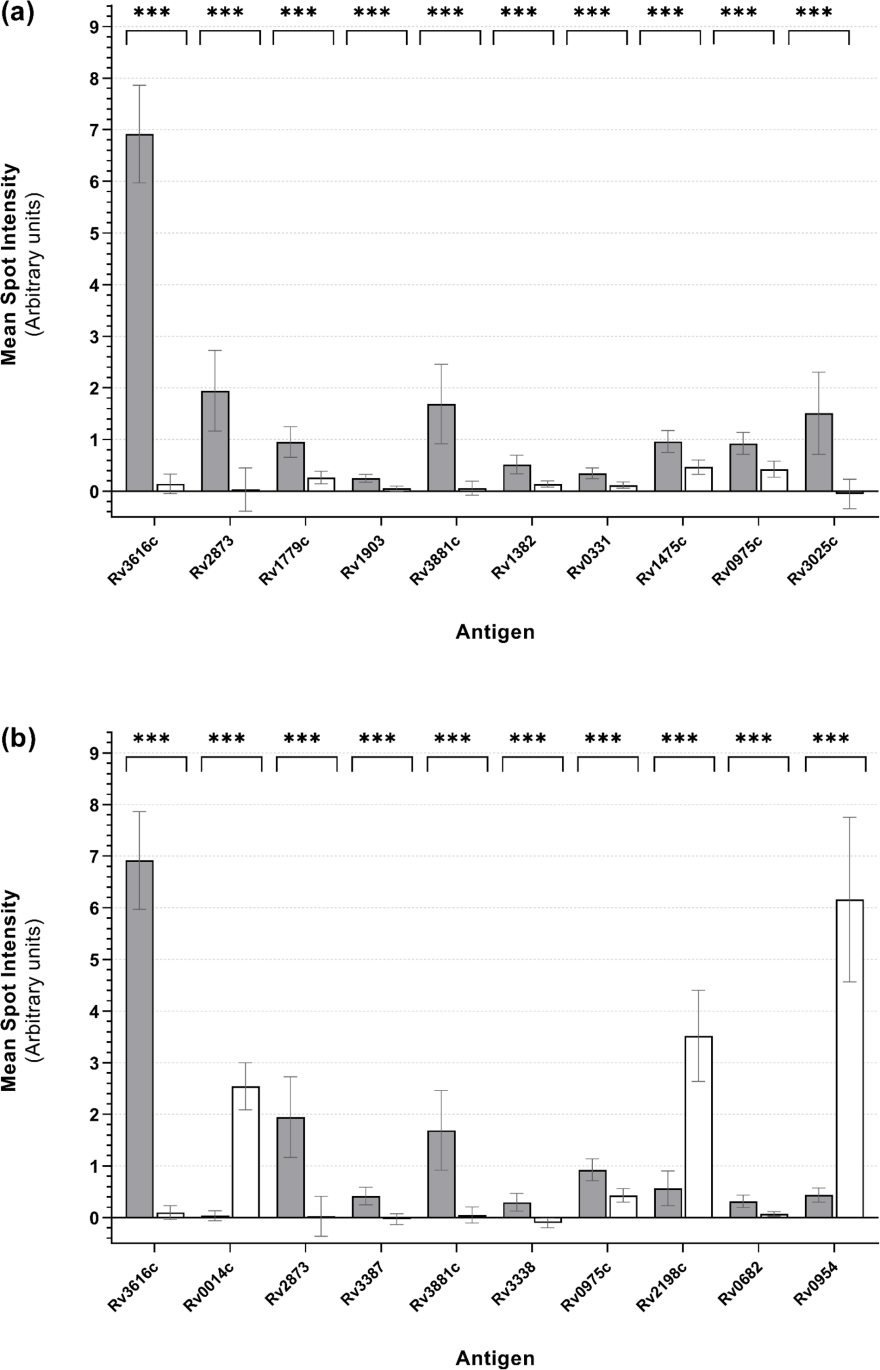
Mean microarray antigen spot intensities from TB-infected and BCG-vaccinated badger serum samples. Gray bars represent sera from TB-infected badgers collected 12–13 weeks after experimental infection; white bars represent sera from badgers that received (**a**) a single BCG vaccination or (**b**) two BCG vaccinations. Mean spot intensities (log₂-normalized) are shown with 95% confidence interval error bars. Independent-samples *t*-tests were used to compare antigen intensities between TB-infected and BCG-vaccinated groups. Antigens were ranked by BH-adjusted *P*-values, and the 10 most differential antigens (defined as those with the lowest BH-adjusted *P*-values) for each comparison group are shown; ***: *P* < 0.001.

Antigens were prioritized by ranking based on *t*-test BH-adjusted *P*-values as an unbiased and systematic approach to guide the selection of antigens with differential mean spot intensity signals consistent with increased antibody binding in sera collected from TB-infected animals. The 10 antigens with the lowest BH-adjusted *P*-values in each comparison group, indicating the most statistically significant differential recognition, are shown in Fig. 2 (TB-free and TB-infected samples, at 8 weeks and at 12–13 weeks post-infection) and Fig. 3 (TB-infected samples at 12–13 weeks post-infection, and samples from BCG-vaccinates that had received one or two vaccine doses). A mean microarray antigen spot intensity of zero indicated that the intensity was no different than that of the control spots (IVTT), and a value of 1.0 (and each additional unit increase thereafter) indicated a doubling of intensity, relative to these controls. The potential serodiagnostic utility of antigens where *t*-test BH-adjusted *P*-values were < 0.05 is summarized in Table 1.

**TABLE 1:** Summary of the discriminative potential of identified antigens^*a*^

Antigen	Comparison groups			
	TB-infected (8 weeks) + TB-free	TB-infected (12–13 weeks) + TB-free	TB-infected (12–13 weeks) + 1× BCG	TB-infected (12–13 weeks) + 2× BCG
Rv0014c	–	–	–	X
Rv0331	–	–	X	–
Rv0584	–	X	–	–
Rv0682	–	–	–	X
Rv0729	X	–	–	–
Rv0954	–	–	–	X
Rv0975c	–	–	X	X
Rv1238	–	X	–	–
Rv1382	–	–	X	–
Rv1400c	–	X	–	–
Rv1475c	–	–	X	–
Rv1530	–	X	–	–
Rv1566c	X	X	–	–
Rv1779c	–	–	X	–
Rv1903	–	–	X	–
Rv2198c	–	–	–	X
Rv2873	–	X	X	X
Rv2875	–	X	–	–
Rv3025c	–	X	X	–
Rv3338	–	–	–	X
Rv3387	–	–	–	X
Rv3616c	X	X	X	X
Rv3881c	X	X	X	X

^*a*^ The potential badger diagnostic utility of differential antigens identified through proteome microarray screening. Antigens are listed in alphanumeric order. An “X” indicates a statistically significant difference (*P* < 0.05) between comparison groups based on *t*-test results.

Mining the MTb H37Rv proteome microarray identified a series of antigens with differential recognition and potential serodiagnostic utility for TB-infected and BCG-vaccinated badgers. Comparison of TB-free and TB-infected animals (12–13 weeks post-infection; Fig. 2a) using the BH-adjusted *P*-value ranking approach identified both Rv2873 (MPB83) and Rv2875 (MPB70), which are the antigens included in the Badger *M. bovis* Ab Test, as significantly differentially recognized. In general, mean spot intensities (see Fig. 2 and 3) for sera from TB-infected animals were greater than those of sera from TB-free or BCG-vaccinated animals for each antigen, but this pattern was reversed for some antigens (most notably Rv0014c, Rv0954, and Rv2198c). Overall, the mean spot intensity results for different comparison groups suggest that some antigens may have broader diagnostic utility than others (see Table 1). For example, Rv3616c and Rv3881c may be able to differentiate not only between TB-free and TB-infected badgers (at both earlier and later stages of infection) but also between TB-infected and BCG-vaccinated animals. Additionally, Rv0014c, Rv0954, and Rv2198c were identified as potential biomarkers of BCG vaccination in badgers; however, the unequal sizes of the comparison groups (TB-infected, *n* = 29; BCG-vaccinated, *n* = 6) and the fact that these groups were independent rather than paired, likely limited the statistical power of the *t*-tests to detect true differences. These findings should therefore be interpreted with caution.

For antigens in comparison groups where the serum panel sample sizes were equal (*n* = 29) and paired *t*-tests could be carried out (Fig. 2), Rv3616c was particularly notable in that it had both the lowest BH-adjusted *P*-value and the highest fold change in mean spot intensity across both time points post-infection of all ranked antigens. Based on exponentiated log₂-transformed values, the mean spot intensity for Rv3616c increased 7.3-fold at 8 weeks and 105.7-fold at 12–13 weeks post-infection for TB-infected samples, relative to TB-free samples.

### ELISA screening of Rv3616c peptides to identify immunodominant diagnostic epitopes

**Fig 4:**
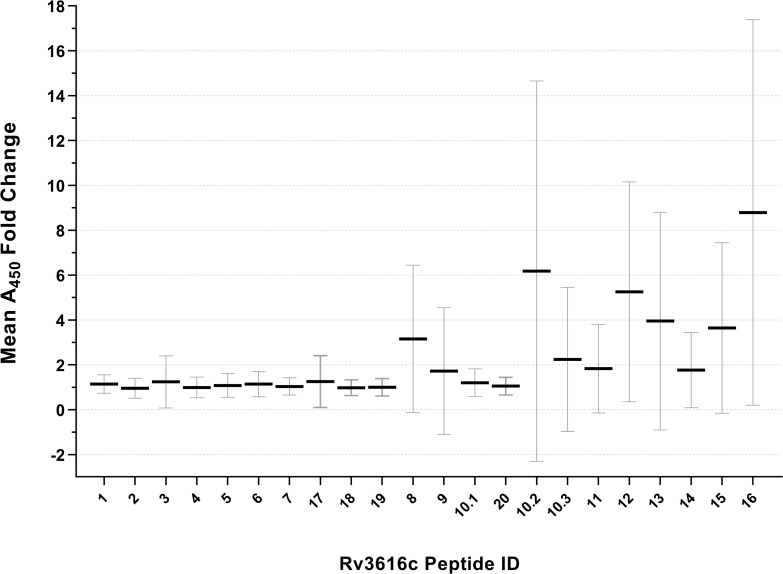
Mean 450 nm absorbance (A_450_) fold changes in Rv3616c peptide ELISA results using serum samples from TB-free and TB-infected badgers. Serum samples from 29 animals, collected before and 12–13 weeks after experimental infection, were tested on Badger Rv3616c ELISA plates, each coated with one of 22 Rv3616c synthetic peptides. For each animal, the post-infection A_450_ value was divided by the corresponding pre-infection value to calculate a fold change. Mean fold-change values for all 29 animals are shown for each individual peptide, with SD error bars. Peptides are ordered along the x-axis according to their starting amino acid positions within the Rv3616c (from N-terminus to C-terminus), rather than by numerical ID reference.

Preliminary diagnostic evaluation of the antigens with potential serodiagnostic utility identified during proteome microarray analysis (shown in Table 1) could not be immediately pursued, as these antigens were not commercially available as proteins. However, we were able to carry out a detailed evaluation of Rv3616c, one of the most diagnostically interesting antigens identified, as it was available to us in the form of 22 individual overlapping synthetic peptides (20–40 amino acids in length) from an unrelated study. As an initial peptide screen, separate Badger Rv3616c ELISA plates were each coated with one of the 22 individual synthetic peptides and used to test the TB-free and TB-infected (12–13 weeks post-infection) serum samples from 29 animals that were part of the original antigen mining serum panel, and the results, expressed as absorbance fold-change values, are shown in Fig. 4. Despite considerable variation in individual animal results (reflected in the magnitude of the standard deviation [SD] error bars), the greatest mean fold change in absorbance results appeared to be achieved using ELISA plates coated with antigens from a region of the Rv3616c protein flanked by synthetic Peptides 8 and 16. These 11 overlapping peptides encompass the continuous region Ile 181 to Val 392 of the Rv3616c protein. Although Peptide 20 (Glu 230 to Thr 250) also falls in this continuous region, albeit with the lowest mean absorbance fold-change value of these eleven synthetic peptides, it was subsequently identified as missing a single amino acid (Phe 233) present in the Rv3616c protein sequence. Fortunately, the correct sequence, which includes Phe 233, was also present within Peptide 10.1 (see Fig. 1).

ROC analyses were carried out on the individual ELISA data obtained for each of the 11 synthetic peptides (Peptides 8, 9, 10.1, 10.2, 10.3, 11, 12, 13, 14, 15, and 16) representing the Ile 181 to Val 392 region of Rv3616c, to identify a smaller, more practical, and diagnostically relevant potential pool of synthetic peptides for further evaluation. The results are summarized in Table 2, and ROC curves are provided in Fig. S1. The highest common test Sp value across these 11 synthetic peptides was 96.55% (95% CI: 82.82–99.82), corresponding to a wide range of individual peptide test sensitivities (10.34%–72.41%) and test cut-off values (>0.09485–1.342).

**TABLE 2:** Test performance of individual Rv3616c peptide ELISAs using serum samples from badgers tested with 11 selected synthetic peptides^*a*^^,^^*b*^

Peptide ID	ROC		Se	
	AUC (95% CI)	AUC diagnostic value	A_450_ cut-off	% Se (95% CI)
8	0.7313 (0.5986–0.8639)	Fair	0.6245	27.59 (14.70–45.72)
9	0.5339 (0.3824–0.6854)	Fail	0.1973	10.34 (3.58–26.39)
10.1	0.5279 (0.3746–0.6813)	Fail	0.1222	10.34 (3.58–26.39)
10.2	0.7919 (0.6753–0.9085)	Fair	0.09485	55.17 (37.55–71.59)
10.3	0.6480 (0.5044–0.7917)	Poor	0.1182	17.24 (7.60–34.55)
11	0.6665 (0.5276–0.8053)	Poor	0.109	31.03 (17.28–49.23)
12	0.8347 (0.7205–0.9490)	Good	0.1207	72.41 (54.28–85.30)
13	0.8347 (0.7233–0.9461)	Good	0.1735	65.52 (47.35–80.06)
14	0.6760 (0.5369–0.8150)	Poor	0.4732	13.79 (5.50–30.56)
15	0.7408 (0.6103–0.8713)	Fair	0.1406	51.72 (34.43–68.61)
16	0.8109 (0.6950–0.9269)	Good	1.342	24.14 (12.22–42.11)

^*a*^ ROC analyses were used to estimate individual peptide-specific test cut-off values and test sensitivities at a fixed test Sp of 96.55% (95% CI: 82.82–99.82), the highest common test Sp value across all 11 synthetic peptides.

^*b*^ CI, confidence interval; A_450_, absorbance at 450 nm; AUC diagnostic value, a qualitative description of the diagnostic discriminatory power of each peptide-based ELISA based on the AUC value (46).

Sera collected from nine TB-free animals recognized at least 1 of each of the 11 peptides, but the sera collected from 20 TB-free animals did not recognize any of the 11 peptides selected. For TB-infected animal sera, Peptide 12 was the most frequently recognized of the 11 peptides selected (21/29 or 72.41% of individual animals), and Peptides 9 and 10.1 were the least frequently recognized (3/29 animals or 10.34% of animals). A combination of four synthetic peptides (Peptides 10.2, 12, 13, and 15) was recognized by 27 out of 29 (93.1%) sera samples from TB-infected animals and by only 4 out of 29 (13.8%) serum samples from TB-free animals. Peptides 12, 13, and 15 encompass a continuous amino acid sequence within Rv3616c (see Fig. 1) that extends from Gly 281 to Ala 380, while Peptide 10.2 is located distally to this region, extending from Gly 241 to Leu 264. The theoretical addition of any further synthetic peptides (from the group of eleven) to this four-peptide combination would not have improved the true positive detection rate, since the sera collected from two TB-infected animals were not recognized by any of the selected 11 synthetic peptides. Consequently, a single four-peptide ELISA (hereafter referred to as the Rv3616c-4P Badger ELISA) was developed, containing synthetic Peptides 10.2, 12, 13, and 15 for extended diagnostic test performance evaluation using a larger number of TB-free and TB-infected badger sera samples.

### ROC analysis and cut-off value determination for the Rv3616c-4P Badger ELISA

Archived serum samples originally collected from 173 TB-free and 98 TB-infected badgers were used to evaluate potential test cut-off values for the Rv3616c-4P Badger ELISA. For ROC analysis, serum samples from both experimentally and naturally infected animals were combined into a single TB-infected group to improve the precision and reliability of cut-off value estimates. The grouping of experimentally and naturally infected animals into a single TB-infected group was statistically supported by nonparametric ANOVA (Kruskal-Wallis test: *P* < 0.0001) followed by Dunn’s multiple comparison tests which showed no significant differences in Rv3616c-4P Badger ELISA absorbance values between the experimentally and naturally infected groups (*P* >0.9999). These results indicated that serum samples from the different TB-infected groups were statistically indistinguishable in terms of their Rv3616c-4P Badger ELISA absorbance values. In contrast, Rv3616c-4P Badger ELISA absorbance values for both infected groups differed significantly from the TB-free group (*P* < 0.0001), indicating increased binding of serum antibodies to the ELISA peptides relative to the TB-free group. This immunological difference demonstrated the ability of the Rv3616c-4P Badger ELISA to effectively differentiate between TB-free and TB-infected serum samples, regardless of whether infection was experimental or natural.

The test performance of the Rv3616c-4P Badger ELISA was evaluated using ROC analysis (ROC curve shown in Fig. S2), resulting in an AUC value of 0.9226 (*P* < 0.0001; 95% CI: 0.8796–0.9655), indicating an excellent ability (47) of this test to distinguish between serum samples collected from TB-free and TB-infected animals. As generally recommended, the optimum cut-off for the Rv3616c-4P Badger ELISA was initially evaluated using several independent methods, in this case, Youden’s J Statistic, Euclidean Distance, Maximum Product, Index of Union, and DOR methods (48, 49). Additionally, potential test cut-off values were determined by selecting those that would generate Se or Sp values that best approximated those of the existing Badger *M. bovis* Ab Test. Each of the resulting Rv3616c-4P Badger ELISA potential test cut-off values and their associated test performance characteristics are summarized in Table 3.

**TABLE 3:** Determination of potential cut-off values for the Rv3616c-4P Badger ELISA^*a*^^,^^*b*^

Method	Cut-off A_450_	% Se (95% CI)	% Sp (95% CI)	% Accuracy (95% CI)
ED	>0.1072	88.78 (81.01–93.62)	91.33 (86.19–94.68)	90.41 (86.26–93.64)
J	>0.1279	85.71 (77.44–91.30)	94.80 (90.41–97.24)	91.51 (87.54–94.54)
MP	>0.1279	85.71 (77.44–91.30)	94.80 (90.41–97.24)	91.51 (87.54–94.54)
IOU	>0.1279	85.71 (77.44–91.30)	94.80 (90.41–97.24)	91.51 (87.54–94.54)
vELISA Sp	>0.2328	74.49 (65.00–82.08)	98.27 (95.03–99.53)	89.67 (85.41–93.02)
vELISA Se	>0.2689	71.43 (61.81–79.43)	98.84 (95.88–99.79)	88.93 (84.58–92.41)
DOR	>0.2737	70.41 (60.74–78.54)	99.42 (96.80–99.97)	88.93 (84.58–92.41)

^*a*^ Potential test cut-off values were derived using a range of different methods, as well as values that would result in a test Se and test Sp approximating those of the Badger *M. bovis* Ab Test. Accuracy was calculated as (Se × Pr) + [Sp × (1 − Pr)], where Pr was the infection prevalence (36.16%) represented by the test sample set.

^*b*^ 95% CI, 95% confidence interval; A_450_, absorbance at 450 nm; ED, Euclidean distance (cut-off at minimum √[(1 − Se)^2^ + (1 − Sp)^2^]; J, Youden’s J statistic (cut-off at maximum Se + Sp − 1); MP, maximum product (cut-off at maximum Se × Sp); IOU, index of union (cut off at minimum (Se − AUC) + (Sp − AUC)); DOR, diagnostic odds ratio (cut off at maximum (Se × Sp) / [(1 - Se) × (1 − Sp)]); vELISA Sp, cut off resulting in an Sp that approximates to that of the Badger *M. bovis* Ab Test (98.52%; 95% CI: 95.75–99.60); vELISA Se, cut-off resulting in an Se that approximates to that of the Badger *M. bovis* Ab Test (71.65%; 95% CI: 63.27–78.77).

Potential cut-off values for the Rv3616c-4P Badger ELISA ranged from >0.1072 to >0.2737. Increasing cut-off values were associated with decreasing test Se (from 88.78% to 70.41%) and increasing Sp (from 91.33% to 99.42%). Although the test accuracy values were broadly similar across the range of cut-off values and their confidence intervals overlapped substantially, a cut-off value of >0.1279 was identified by three independent determination methods and resulted in the highest estimated test accuracy (91.51%). This threshold was therefore selected for subsequent direct comparison of the Rv3616c-4P Badger ELISA and the Badger *M. bovis* Ab Test.

### Comparative performance of the Rv3616c-4P Badger ELISA and the Badger *M. bovis* Ab Test

Table 4 summarizes the diagnostic performance metrics obtained for both tests (Badger *M. bovis* Ab Test: cut-off >1.674; Rv3616c-4P Badger ELISA: cut-off >0.1279) with corresponding 95% confidence intervals. Overall accuracy was similar for both tests: 89.30% (95% CI: 84.99–92.72) for the Badger *M. bovis* Ab Test and 91.51% (95% CI: 87.54–94.54) for the Rv3616c-4P Badger ELISA. The Badger *M. bovis* Ab Test had higher Sp (98.27%; 95% CI: 95.02–99.64) than the Rv3616c-4P Badger ELISA (94.80%; 95% CI: 90.35–97.59), while Se was higher for the Rv3616c-4P Badger ELISA (85.71%; 95% CI: 77.19–91.96) than for the Badger *M. bovis* Ab Test (73.47%; 95% CI: 63.59–81.88).

**TABLE 4:** Comparative performance of the Badger *M. bovis* Ab Test and Rv3616c-4P Badger ELISA^*a*^^,^^*b*^

Test	% Se (95% CI)	% Sp (95% CI)	% Accuracy (95% CI)	DOR (95% CI)
Badger *M. bovis* Ab Test	73.47 (63.59–81.88)	98.27 (95.02–99.64)	89.30 (84.99–92.72)	156.56 (46.03–534.98)
Rv3616c-4P Badger ELISA	85.71 (77.19–91.96)	94.80 (90.35–97.59)	91.51 (87.54–94.54)	109.87 (45.45–262.99)

^*a*^ The same 271 serum sample set (173 TB-free and 98 TB-infected) was tested using both the badger *M. bovis* Ab Test (cut-off: A_450_ >1.674) and the Rv3616c-4P Badger ELISA (cut-off: A_450_ >0.1279).

^*b*^ 95% CI, 95% confidence interval; A_450_, absorbance at 450 nm; Test accuracy, Acc., accuracy calculated as (Se × Pr) + [Sp × (1 − Pr)], where Pr is the infection prevalence (36.16%) represented by the test sample set; DOR, diagnostic odds ratio (calculated as (Se × Sp) / [(1 − Se) × (1 − Sp)]).

The DOR integrates Se and Sp into a single metric, providing an overall indicator of diagnostic performance (50), and was included in the comparative analyses because it enabled a direct comparison between tests using one comprehensive measure, rather than separate Se and Sp values. Higher DOR values indicate better test performance, reflecting the ability to discriminate between positive and negative samples. A DOR value of 10 is considered indicative of very good diagnostic performance (51), and both tests exceeded this threshold: the Badger *M. bovis* Ab Test had a DOR of 156.56 (95% CI: 46.03–534.98), and the Rv3616c-4P Badger ELISA had a DOR of 109.87 (95% CI: 45.45–262.99).

Although some apparent differences in diagnostic metrics were observed, the broad and overlapping confidence intervals indicated uncertainty in the precision of these estimates. Formal statistical tests did not detect significant differences (McNemar’s test for paired proportions, *P* = 0.405; *z*-test on natural log-transformed DOR, *P* = 0.645), although the wide confidence intervals associated with these metric estimates may have limited the ability to do so, even if true differences existed (52).

### Diagnostic performance of parallel testing with the Badger *M. bovis* Ab Test and Rv3616c-4P Badger ELISA

Although the differences in performance metrics observed during stand-alone testing were not statistically significant, the Rv3616c-4P Badger ELISA appeared to have superior test Se, while the Badger *M. bovis* Ab Test, which utilizes MPB70 and MPB83 antigens (53), had higher Sp. These observed differences may still be of practical relevance. Consequently, the results of both tests were further evaluated using a parallel interpretation, a strategy that can enhance overall diagnostic performance by combining the strengths of each test (54). This approach can typically increase overall Se with only a modest reduction in Sp, provided each individual test already has relatively high Sp. The diagnostic outcomes of parallel testing were assessed across the range of potential cut-off values previously identified (see Table 3) for stand-alone Rv3616c-4P Badger ELISA testing, while a constant test cut-off was maintained for the Badger *M. bovis* Ab Test. The results of parallel testing using these two tests are summarized in Table 5.

**TABLE 5:** Parallel testing results of the Badger *M. bovis* Ab Test and Rv3616c-4P Badger ELISA^*a*^^,^^*b*^

Rv3616c-4P Badger ELISA	TP	FN	FP	TN	Parallel testing % Se (95% CI)	Parallel testing % Sp (95% CI)
Cut-off						
>0.1072	91	7	18	155	92.86 (85.84–97.08)	89.60 (84.06–93.72)
>0.1279	90	8	12	161	91.84 (84.55–96.41)	93.06 (88.20–96.36)
>0.2328	85	13	6	167	86.73 (78.38–92.74)	96.53 (92.60–98.72)
>0.2689	82	16	5	168	83.67 (74.84–90.37)	97.11 (93.38–99.06)
>0.2737	82	16	4	169	83.67 (74.84–90.37)	97.69 (94.19–99.37)

Badger *M. bovis* Ab Test	TP	FN	FP	TN	Stand-alone testing % Se (95% CI)	Stand-alone testing % Sp (95% CI)
Cut-off						
>1.674	72	26	3	170	73.47 (63.59–81.88)	98.27 (95.02–99.64)

^*a*^ A total of 271 serum samples (173 TB-free and 98 TB-infected) were tested using both assays. Stand-alone testing results using just the Badger *M. bovis* Ab Test are included for comparison. A fixed cut-off value of >1.674 was used for the Badger *M. bovis* Ab Test as it is a validated diagnostic test for TB in badgers. A range of potential cut-off values was applied to the Rv3616c-4P Badger ELISA, including the previously identified stand-alone test cut-off value of >0.1279, to evaluate the impact on test result classification under a parallel testing interpretation. These cut-off values were derived using the following methods (see also Table 3): 0.1072: Euclidean Distance; 0.1279: Youden’s J Statistic, Maximum Product and Index of Union; 0.2328: Sp-matched to the Badger *M. bovis* Ab Test; 0.2689: Se-matched to the Badger *M. bovis* Ab Test; 0.2737: DOR.

^*b*^ TP, true positive; FN, false negative; FP, false positive; TN, true negative; 95% CI, 95% confidence interval.

Compared to the stand-alone use of the Badger *M. bovis* Ab Test, which correctly identified 72 of 98 true positive samples (Se: 73.47%; 95% CI: 63.59–81.88), all parallel testing combinations evaluated resulted in improved Se. This improvement, however, was accompanied by varying trade-offs in Sp relative to stand-alone use of the Badger *M. bovis* Ab Test, depending on the Rv3616c-4P Badger ELISA cut-off value selected. The lowest Rv3616c-4P Badger ELISA cut-off evaluated (>0.1072) resulted in the highest Se (92.86%; 95% CI: 85.84–97.08), but this was accompanied by a decrease in Sp (89.60%; 95% CI: 84.06–93.72), compared to stand-alone use of the Badger *M. bovis* Ab Test (98.27%; 95% CI: 95.02–99.64). At the highest Rv3616c-4P Badger ELISA cut-off value assessed (>0.2737), an overall testing Sp, or true negative rate, of 97.69% (95% CI: 94.19–99.37) was achieved, which closely matched the value of 98.27% (95% CI: 95.02–99.64) observed with stand-alone use of the Badger *M. bovis* Ab Test. Moreover, using this cut-off and a parallel interpretation resulted in an overall testing Se, or true positive rate, of 83.67% (95% CI: 74.84–90.37) compared to 73.47% (95% CI: 63.59–81.88) for the Badger *M. bovis* Ab Test when used in isolation. In practical terms, with this test sample set, a parallel testing approach using an Rv3616c-4P Badger ELISA cut-off value of >0.2737 resulted in 10 additional samples collected from TB-infected badgers being classified as positive compared to the Badger *M. bovis* Ab Test alone. This improvement was associated with only one additional sample collected from a TB-free badger being classified as positive (i.e., one additional false positive).

## DISCUSSION

The diagnosis of *M. bovis* infection in badgers remains challenging, with the effectiveness of current serological tests for surveillance and disease control constrained by their limited performance. Proteome microarray technology is becoming an increasingly valuable tool for the identification of potentially novel diagnostic biomarkers of infection, investigating disease pathogenesis, and supporting vaccine development (55–58). It has been successfully used to identify new serodiagnostic targets of mycobacterial infections in humans and animals (59–61). Using proteome-scale antigen discovery and an ELISA-based epitope mapping strategy, we identified four individual peptide sequences (comprising 124 non-overlapping amino acids) within the C-terminal region of Rv3616c (Gly 241 to Ala 380) with serodiagnostic potential. When combined as a peptide pool (Rv3616c-4P) and used as the coating antigen in an indirect ELISA, this antigen pool achieved a promising overall diagnostic accuracy of 91.51% (using a stand-alone test cut-off value of >0.1279). This accuracy compared favorably with that of the existing Badger *M. bovis* Ab Test (89.30%), which contains *M. bovis* antigens MPB83 (Rv2873) and MPB70 (Rv2875) (53).

Parallel testing can sometimes offer improved diagnostic performance compared to relying on a single test, particularly when individual test specificities are already high. In this study, we demonstrated that applying a parallel interpretation to the results of both the Badger *M. bovis* Ab Test and the Rv3616c-4P Badger ELISA (using a parallel testing cut-off value of >0.2737) resulted in an increase in Se, as expected with parallel testing, with only a minimal decrease in Sp. This combined approach correctly classified more true positive samples than the Badger *M. bovis* Ab Test alone, with little impact on the true negative detection rate. As with all diagnostic tests, cut-off values must ultimately be optimized for each test to ensure that they meet the required thresholds for positive predictive values (PPV) and negative predictive values (NPVs) under different disease prevalence scenarios (62). The relative importance of PPV and NPV depends on the specific objectives of a testing program. For example, in lower-prevalence settings, PPV could be prioritized to minimize the risk of inappropriate badger TB control interventions, such as unnecessary culling or misdirected vaccination (e.g., vaccination of animals already infected with TB, because BCG is most effective in badgers when used prophylactically). Conversely, NPV could be more important in a Test and Vaccinate or Remove (TVR) badger TB control strategy where it would help to ensure that only uninfected badgers are released after trapping.

Figure S3 illustrates how disease prevalence might influence the PPV, NPV, and overall test accuracy of the Badger *M. bovis* Ab Test, the Rv3616c-4P Badger ELISA (shown using two example cut-off values), and parallel testing strategies. For context, a recent study estimated an overall TB prevalence of 6.5% (range: 1.1%–13.0%) in badgers (found dead) within the UK southern edge area of the bTB epidemic, into which the disease is expanding (9). If these tests were to be applied in this region of the UK, stand-alone testing with the Rv3616c-4P Badger ELISA (using a higher cut-off value) would be expected to provide the greatest PPV, reducing the likelihood of false positives. In contrast, parallel testing combining the Badger *M. bovis* Ab Test with the Rv3616c-4P Badger ELISA (at a lower cut-off value) would likely provide the highest NPV, minimizing the risk of false negatives. This illustrative scenario highlights the importance of context-specific test calibration when deploying badger serodiagnostic tools for TB surveillance and control, ensuring that they are optimally aligned with relevant epidemiological settings and testing program objectives (52).

Mining the *M. tuberculosis* proteome (rather than the *M. bovis* proteome) using serum samples collected from badgers experimentally infected with *M. bovis* (as opposed to those from naturally infected badgers) was an accepted limitation of this study. Although *M. tuberculosis* and *M. bovis* share extensive genomic and protein-coding gene similarity (36), and experimental infection has been shown to closely mimic natural infection in badgers (63), it remains possible that some serodiagnostic antigen targets unique to natural *M. bovis* infection in badgers may not have been identified using this approach. Additionally, a statistical ranking approach was used in this study to facilitate rapid identification and prioritization of antigens for evaluation in a serodiagnostic test format. Although this approach is commonly used and generally accepted during early-stage proteome microarray antigen discovery, it ranks antigens based solely on statistical significance between comparison groups. Consequently, antigens with potential biological relevance but borderline or weak statistical support may have been overlooked.

Despite these limitations, the comprehensive proteome microarray antigen screening and statistical ranking strategies applied in this study identified MPB83 (Rv2873) and MPB70 (Rv2875), which are present in the existing APHA Badger *M. bovis* Ab Test for TB in badgers, supporting the suitability of the antigen discovery and ranking approaches used. These strategies also identified several novel badger TB serodiagnostic targets for further investigation, including the protein product of the *Rv3616c* gene. Additional antigen targets were identified as part of the comprehensive screening and ranking strategies applied in this study, including several others that could be used in tests that are able to differentiate infected from vaccinated animals (DIVA tests) and putative biomarkers of BCG vaccination, but their evaluation was beyond the scope of the present study. These antigens represent potential targets for future research aimed at improving diagnostic tools or monitoring vaccination as part of badger TB surveillance.

The protein product of the *Rv3616c* gene in *M. tuberculosis* (or *Mb3646c* in *M. bovis*), ESX-1 secretion-associated protein A, is a major secretory protein involved in mycobacterial virulence and disease pathogenesis (64). Rv3616c has previously been identified as an immunodominant antigen in cattle infected with *M. bovis* (65) and could potentially form part of a molecularly defined tuberculin replacement for the existing purified protein derivatives used in the primary diagnostic screening test for bTB in UK cattle (the single intradermal comparative cervical tuberculin test) (66). The existing commercial diagnostic tests for TB infection in badgers include the DPP VetTB Assay, which uses antigens MPB83 and ESAT6-CFP10, and the Badger *M. bovis* Ab Test, a modified version of the IDEXX *M. bovis* Ab Test for cattle that uses antigens MPB70 and MPB83 (26, 53). The Rv3616c-4P peptide pool identified in this study represents a promising combination of novel serodiagnostic antigens that could be incorporated into a new diagnostic test or combined with existing antigens to improve the performance of current serological assays for TB in badgers. Peptide-based antigens are particularly attractive because they can be synthetically manufactured at scale, ensuring high purity at a relatively low cost.

The Badger *M. bovis* Ab Test consistently performed well in this study and has been recently validated by APHA as a diagnostic test for TB in badgers. The data obtained using the Badger *M. bovis* Ab Test and the Rv3616c-4P Badger ELISA in parallel testing support the proposition that a diagnostic test for TB in badgers incorporating Rv3616c-4P, MPB70, and MPB83 could deliver improved accuracy and provide maximum flexibility of use according to disease prevalence and testing objectives, such as prioritizing PPV or NPV. Such a test could enhance Se while retaining the Sp of the Badger *M. bovis* Ab Test and therefore merits further investigation.

### Conclusion

Badgers are a wildlife reservoir for *M. bovis* in the UK and represent a source of both direct and indirect transmission to cattle. Addressing TB infection in badgers is therefore a critical component of efforts to eradicate bTB from cattle herds. In recent years, this has included government-licensed badger culls aimed at reducing transmission risk, although these were not based on the infection status of individual animals. Badger vaccination with BCG has increasingly been implemented as a non-lethal TB control measure to mitigate infection risks to cattle. As part of a refreshed TB eradication strategy, the UK government is phasing out culling, increasing support for badger vaccination, and enhancing TB surveillance in wildlife. The development of novel serodiagnostic antigens for detecting TB in badgers could enhance diagnostic accuracy and strengthen epidemiological surveillance of this key wildlife host of *M. bovis* in the UK.

Peptide-based epitope mapping of the C-terminal region of the Rv3616c protein, identified through comprehensive and unbiased antigen discovery, has revealed promising serodiagnostic targets for detecting TB infection in badgers. If successfully validated for laboratory use or developed into a rapid, accurate, and user-friendly POCT, these antigens could improve TB surveillance in badger populations and better inform disease control policies compared to existing serological diagnostic tests. Improving surveillance and reporting of TB in wildlife reservoirs is a key priority outlined in the Roadmap for Zoonotic Tuberculosis (67), jointly developed by the World Health Organization (WHO), Food and Agriculture Organization of the United Nations (FAO), World Organisation for Animal Health (WOAH), and the International Union Against Tuberculosis and Lung Disease (The Union). This roadmap aligns with and reinforces the broader One Health approach to global health, supporting the WHO End TB Strategy’s vision of a world free of TB (68).

## Data Availability

The summary proteome microarray and diagnostic testing data generated and analyzed during this study are presented within the article. However, for commercial reasons, the detailed data associated with these studies are confidential and have not been deposited in a publicly accessible repository.

## SUPPLEMENTAL MATERIAL

Supplemental figures and table jcm.01260-25-s0001.pdf Figures S1 to S3, and Table S1.

ASM does not own the copyrights to Supplemental Material that may be linked to, or accessed through, an article. The authors have granted ASM a non-exclusive, world-wide license to publish the Supplemental Material files. Please contact the corresponding author directly for reuse.
