# Supplementary material for: Proteome microarray-guided identification of mycobacterial antigens and ELISA-based peptide mapping for improved serological detection of Mycobacterium bovis infection in European badgers
Source: J Clin Microbiol. 2026 Jan 27;64(3):e01260-25. doi: 10.1128/jcm.01260-25 (PMC12977535; doi:10.1128/jcm.01260-25)
Supplement: Supplemental figures and table — Figures S1 to S3, and Table S1. [file jcm.01260-25-s0001.pdf]

Supplemental Material for "Proteome microarray-guided identification of mycobacterial antigens and ELISA-based peptide mapping for improved serological detection of *Mycobacterium bovis* infection in European badgers"

Table of Contents

Supplemental Figure Legends: Page 3

Supplemental Tables: Page 4

Supplemental Figures: Pages 5-7

Supplemental Figures Legends

Figure S1. Receiver Operating Characteristic (ROC) curves from Rv3616c peptide ELISA results. ROC curves were calculated from ELISA absorbance values (measured at 450 nm) obtained for each of the eleven Rv3616c synthetic peptides, using serum samples collected from 29 TB-free and 29 TB-infected badgers (12–13 weeks after experimental infection).

Figure S2. Receiver Operating Characteristic (ROC) curve from the Rv3616c-4P Badger ELISA results. The ROC curve was calculated from ELISA absorbance values (measured at 450 nm) obtained for 173 TB-free and 98 TB-Infected (26 experimentally infected and 72 naturally infected) serum samples.

Figure S3. Estimated (a) positive predictive value (PPV), (b) negative predictive value (NPV), and (c) accuracy as functions of disease prevalence for the Badger *M. bovis* Ab Test, the Rv3616c-4P Badger ELISA and parallel test interpretations (illustrative figure only, confidence intervals intentionally omitted for clarity). PPV, NPV, and accuracy graph lines were calculated based on laboratory testing results for 173 TB-negative and 98 TB-positive badgers (26 experimentally infected and 72 naturally infected) across a range of simulated disease prevalences. The vertical marker line on the x-axis indicates the estimated overall badger TB prevalence (6.5%) across five counties on the southern edge of England’s bovine TB (bTB) epidemic (9).

<u>Figure S3. Key:</u>

A, Badger *M. bovis* Ab Test at a cut off A450 >1.674;

B, Rv3616c-4P Badger ELISA at cut off A450 >0.1279;

C, Rv3616c-4P Badger ELISA at cut off A450 >0.2737;

D, Parallel interpretation of A and B;

E, Parallel interpretation A and C.

Supplemental Tables

Table S1. Amino acid sequences of individual purified synthetic Rv3616c peptides. The overlapping peptides, ranging from 20 to 40 amino acids in length, collectively span the full 392-amino-acid sequence of the Rv3616c protein (ESX-1 secretion-associated protein A), the product of the *Mycobacterium tuberculosis* H37Rv *espA* gene.

Synthetic Peptide ID	Synthetic Peptide Amino Acid Sequence
P1	MSRAFIIDPTISAIDGLYDLLGIGIPNQGGILYSSLEYFE
P2	LGIGIPNQGGILYSSLEYFEKALEELAAAFPGDGWLGSAA
P3	KALEELAAAFPGDGWLGSAADKYAGKNRNHVNFFQELADL
P4	DKYAGKNRNHVNFFQELADLDRQLISLIHDQANAVQTTRD
P5	DRQLISLIHDQANAVQTTRDILEGAKKGLEFVRPVAVDLT
P6	ILEGAKKGLEFVRPVAVDLTYIPVVGHALSAAFQAPFCAG
P7	YIPVVGHALSAAFQAPFCAGAMAVVGGALAYLVVKTLINA
P8	IISDVADIIKGTLGEVWEFITNALNGLKELWDKLTGWVTG
P9	TNALNGLKELWDKLTGWVTGLFSRGWSNLESFFAGVPGLT
P10.1	LESFFAGVPGLTGATSGLSQVTGL
P10.2	GATSGLSQVTGLFGAAGLSASSGL
P10.3	FGAAGLSASSGLAHADSLASSASL
P11	SSGLAHADSLASSASLPALAGIGGGSGFGGLPSLAQVHAA
P12	GIGGGSGFGGLPSLAQVHAASTRQALRPRADGPVGAAAEQ
P13	STRQALRPRADGPVGAAAEQVGGQSQLVSAQGSQGMGGPV
P14	VGGQSQLVSAQGSQGMGGPVGMGGMHPSSGASKGTTTKKY
P15	GMGGMHPSSGASKGTTTKKYSEGAAAGTEDAERAPVEADA
P16	KGTTTKKYSEGAAAGTEDAERAPVEADAGGGQKVLVRNVV
P17	AMAVVGGALAYLVVKTLINATQLLK
P18	VKTLINATQLLKLLAKLAELVAAAI
P19	LAKLAELVAAAIADIISDVADIIKG
P20	ESFAGVPGLTGATSGLSQVT

Figure S1.

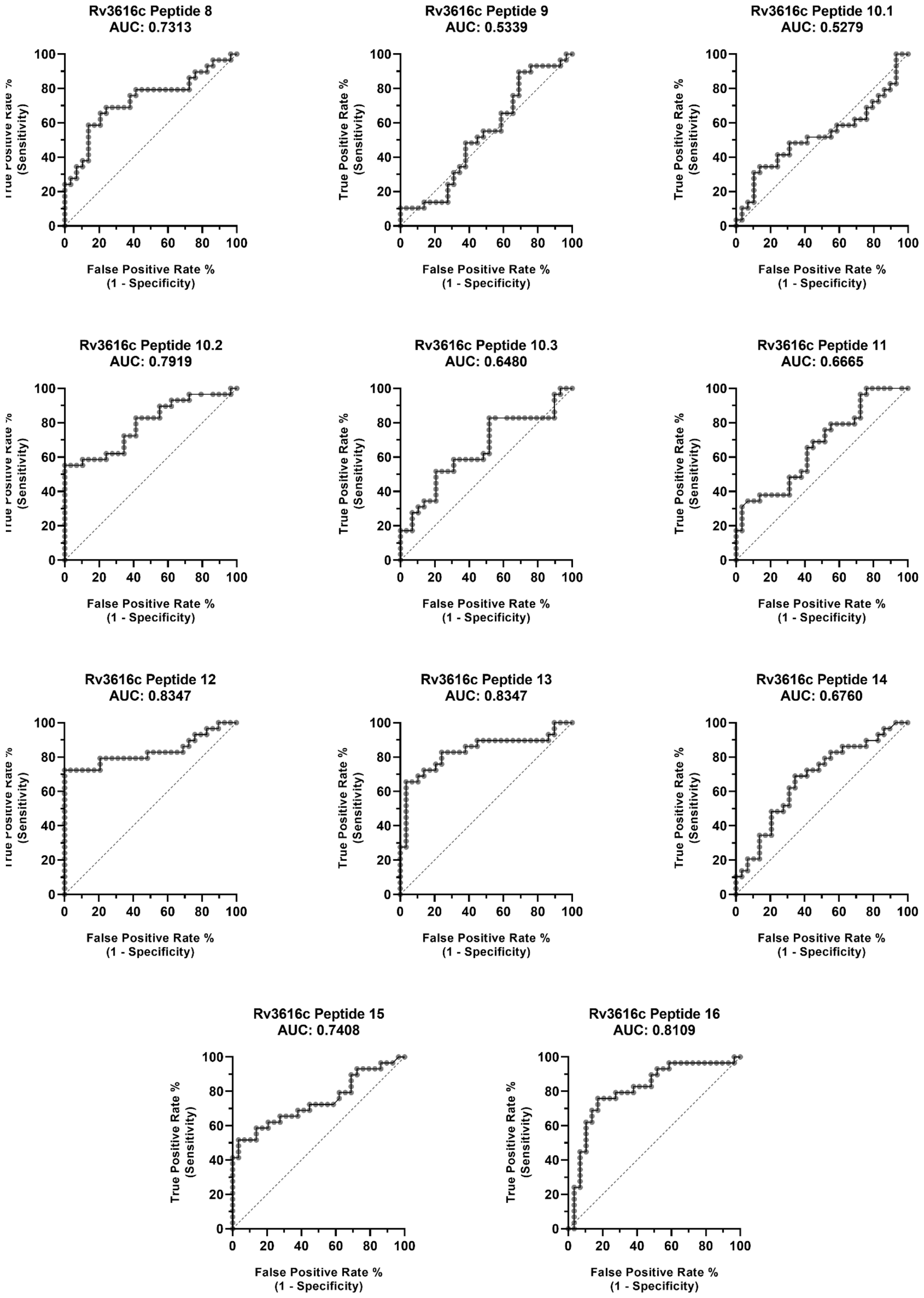
Rv3616c Peptide 8
AUC: 0.7313
Rv3616c Peptide 9
AUC: 0.5339
Rv3616c Peptide 10.1
AUC: 0.5279
Rv3616c Peptide 10.2
AUC: 0.7919
Rv3616c Peptide 10.3
AUC: 0.6480
Rv3616c Peptide 11
AUC: 0.6665
Rv3616c Peptide 12
AUC: 0.8347
Rv3616c Peptide 13
AUC: 0.8347
Rv3616c Peptide 14
AUC: 0.6760
Rv3616c Peptide 15
AUC: 0.7408
Rv3616c Peptide 16
AUC: 0.8109
True Positive Rate %
(Sensitivity)
False Positive Rate %
(1 - Specificity)
0
20
40
60
80
100

Figure S2.

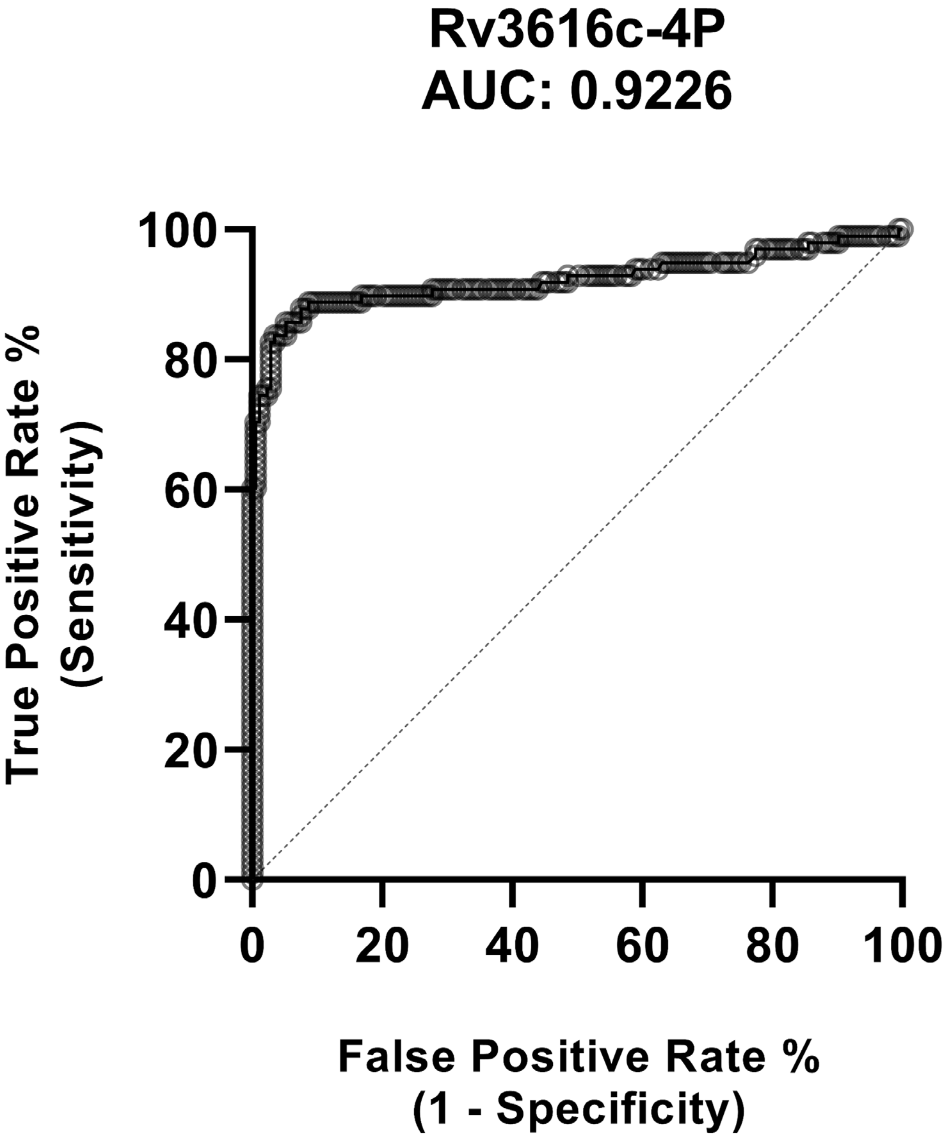
Rv3616c-4P
AUC: 0.9226
100
80
60
40
20
0
True Positive Rate %
(Sensitivity)
0
20
40
60
80
100
False Positive Rate %
(1 - Specificity)

Figure S3.

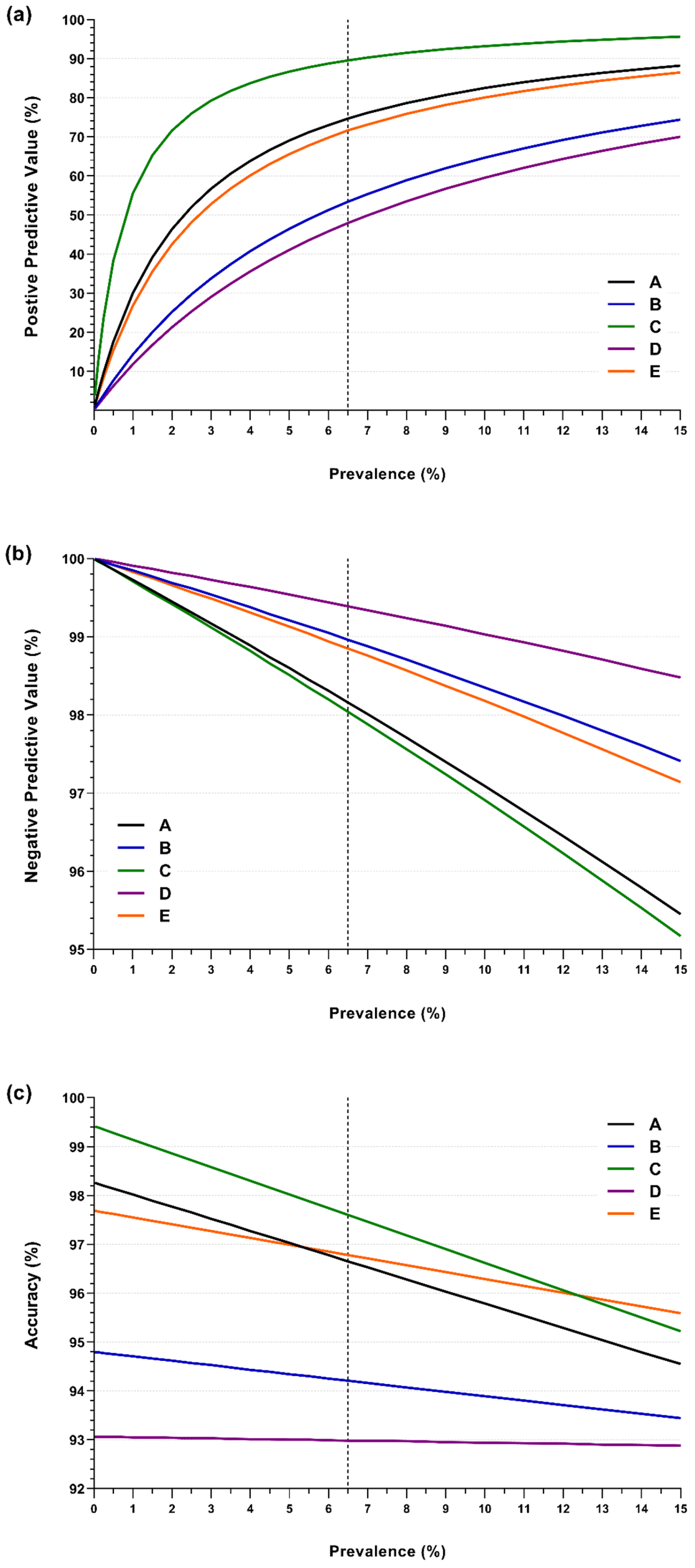
(a)
Postive Predictive Value (%)
Prevalence (%)
A
B
C
D
E
(b)
Negative Predictive Value (%)
Prevalence (%)
A
B
C
D
E
(c)
Accuracy (%)
Prevalence (%)
A
B
C
D
E
